# Socioeconomic inequities and hepatitis A virus infection in Western Brazilian Amazonian children: spatial distribution and associated factors

**DOI:** 10.1186/s12879-015-1164-9

**Published:** 2015-10-16

**Authors:** Saulo A. S. Mantovani, Breno Matos Delfino, Antonio C. Martins, Humberto Oliart-Guzmán, Thasciany M. Pereira, Fernando L. C. C. Branco, Athos Muniz Braña, José A. Filgueira-Júnior, Ana P. Santos, Rayanne A. Arruda, Andréia S. Guimarães, Alanderson A. Ramalho, Cristieli Sergio de Menezes Oliveira, Thiago S. Araújo, Nancy Arróspide, Carlos H. M. L. Estrada, Cláudia T. Codeço, Mônica da Silva-Nunes

**Affiliations:** Centro de Ciências da Saúde e do Desporto, Universidade Federal do Acre. Campus Universitário, BR 364, Km 04, Bairro Distrito Industrial, Rio Branco, AC Brazil; Dirección Regional de Salud de Madre de Dios, Av. Ernesto Rivero N° 475, Puerto Maldonado, Peru; Instituto Nacional de Salud, Cápac Yupanqui 1400 - Jesus María, Lima, 11 Peru; Scientific Computing Program, Avenida Brasil, 4365, Manguinhos, Rio de Janeiro, RJ Brazil

**Keywords:** Hepatitis A, Socioeconomic inequities, Serology, Spatial distribution, Amazon

## Abstract

**Background:**

Hepatitis A is still a neglected health problem in the world. The most affected areas are the ones with disadvantaged socioeconomic conditions. In Brazil, seroprevalence studies showed that 64.7 % of the general population has antibodies against HAV (hepatitis A virus), and the Amazon region has the highest seroprevalence in the country.

**Methods:**

In the present study the seroprevalence of total HAV antibodies in children between 1 and 5 years old residing in the urban area of Assis Brasil, Acre was measured and spatial distribution of several socioeconomic inequities was evaluated.

**Results:**

In the year of 2011, seroprevalence rate was 16.66 %. Factors associated with having a positive serology identified by multivariate analysis were being of indigenous ethnicity [adjusted Odds Ratio (aOR) = 3.27, CI 1.45–7.28], usage of water from the public system (aOR = 8.18, CI 1.07–62.53), living in a house not located in a street (aOR = 3.48, CI 1.54–7.87), and child age over 4 years old (aOR = 2.43, CI 1.23–4.79). The distribution of seropositive children was clustered in the eastern part of the city, where several socioeconomic inequities (lack of flushed toilets, lack of piped water inside the household and susceptibility of the household to flooding during rain, low maternal education, having wood or ground floor at home, and not owning a house, lack of piped water at home, and type of drinking water) also clustered.

**Conclusions:**

The findings highlight that sanitation and water treatment still need improvement in the Brazilian Amazon, and that socioeconomic development is warranted in order to decrease this and other infectious diseases.

**Electronic supplementary material:**

The online version of this article (doi:10.1186/s12879-015-1164-9) contains supplementary material, which is available to authorized users.

## Background

Hepatitis A virus (HAV) is a health problem that is still neglected in several areas in the world, despite the fact that its epidemiology and risk factors are well known. In Brazil, seroprevalence studies showed that 64.70 % of the general population has antibodies against HAV, and the Amazon region has the highest seroprevalence in the country [1]. Clemens et al. [1] compared seroprevalence in four different regions of Brazil, showing a rate of approximately 55.00 % in Southern Brazil and 92.80 % in the Northern region, where the Amazon is located.

In the Western Brazilian Amazon, seroprevalence rates above 90.00 % have been reported for riverine and periurban populations in Acre and Amazonas states [1, 2] and 74.60 % for rural area [3]. In the state of Acre, the incidence of hepatitis A in 2010 was approximately 14 cases per 100,000 inhabitants, the fourth highest in the country [4].

Infection occurs early in life, affecting mainly children between 5 and 9 years of age [4]. In the Amazon, seroprevalence reaches 80.00 % before the age of 10 [5]. Mortality rates for HAV in the Amazon were 0.70 per 100,000 inhabitants in 2010, the highest rate in Brazil [4]. In South Brazil, HAV was responsible for 39.00 % of children’s hospital admissions because of hepatic failure between 1995 and 2006, with a fatal outcome in 69.00 % of the cases.

The reasons seroprevalence for HAV is so high in the Brazilian Amazon are probably related to poor socioeconomic conditions, which tend to be lower in the Amazon region. Most of the Amazonian cities have the lowest human development index (HDI) in Brazil, an index that considers wealth, education, and health as proxies of human development [6]. Some cities in the Northern region have the lowest HDI in Brazil; of 5565 cities in the country, 446 are located in the Northern area and 310 are in the Amazon region. Of these, approximately 54.20 % are in the lowest quartile for the HDI in Brazil [6].

Because most cases in children are asymptomatic, clinical surveillance may not detect transmission areas properly; therefore, the spatial analysis of cases with positive serology may aid in transmission control. Only three published studies have analyzed the spatial distribution of hepatitis A and antibodies prevalent in Brazil. A recent study attempted to analyze spatial distribution of seroprevalence against HAV in a rural area in the Amazon and no spatial clustering was found, but this study was conducted in the general population [7]. Here, the results are observed of a seroprevalence study for HAV antibodies in children under 5 years of age residing in a small town in the Western Brazilian Amazon, as well as the associated factors and the spatial distribution of seropositive cases and social inequities.

## Methods

### Study area

Assis Brasil, located in the state of Acre, was founded in 1976 from old, established communities surrounding rubber plantations. It is located 344 km southwest of Rio Branco, and it borders the municipality of Brazil to the east, the cities of Iñapari (Peru) and Bolpebra (Bolivia) to the south, and the municipality of Sena Madureira to the north. A map showing the location of the city has been published elsewhere [8]. The climate is equatorial hot and humid, a subdivision of tropical climate [9]. In 2003, the population was estimated at 3,667 inhabitants, of whom 62.00 % lived in urban areas. As of early 2011, it had a population of 6,075 inhabitants (3,057 males and 2,960 females), of whom 12.76 % were aged zero to four years. The population that resided in urban areas was estimated at 61.00 % in 2010 [10]. The HDI of Assis Brasil was 0.588 in 2010, being in the lowest quartile among all Brazilian cities [6].

### Study design and population

The population investigated consisted of all children between 12 and 59 months old living in the urban areas of Assis Brazil, in the state of Acre, Brazil. The children were located using the census records of the local community health workers. The location and elevation of all households were determined using a hand-held eTrex global positioning system (Garmin International, Olathe, USA), which gives a positional accuracy within five metres.

Data was collected between January and February 2011, through interviews using structured questionnaires. The following groups of variables were considered: family demographic and socioeconomic conditions; domestic environment conditions; peri-domestic environmental conditions; water source and quality; child demographic information; type and quality of parental care; and access to daycare.

### Sample collection and antibody detection

Venous blood was collected in sterile vacuum tubes with a clot activator. Samples were centrifuged, and the sera were separated and stored in −20 °C until tested. Serum samples were tested for total Ig antibodies to Hepatitis A virus using ELISA commercial kits (Diasorin ETI-AB-HAVK Plus, Diasorin, Italy), following manufacturer´s instruction. Samples were classified as either positive or negative for the presence of antibodies against HAV.

### Statistical analysis

A database was created with SPSS 13.0 software (SPSS Inc., Chicago, IL). The distribution of the independent variables was identified using Student’s *t*-test to compare means, and chi-square test for comparing frequencies or proportions with α = 0.05 critical level. The overall prevalence was calculated.

Exploratory univariate logistic regression analysis was performed using R software version 2.14.0 (The R Foundation for Statistical Computing) [11]. For continuous variables, logistic additive models (gam), we used to verify the assumption of linearity. If not linear, discretization of the variable was done, by cutting at the point that minimized AIC (Akaike Information Criterion). Covariates were maintained in subsequent multivariate logistic regression models, if they were associated with the outcome in the unadjusted analysis at a 20.00 % level of significance. Variables included in each final generalized logistic regression model are shown in the results section. Variable selection was done by comparing AIC. In the presence of collinearity, one of the variables of chosen, also using AIC. To investigate deviation from the independence assumption of glm models, spatial correlograms of residuals from the null model and the final model were inspected using the ncf library of R [12]. This analysis indicates that the proposed model structure was sufficient to account for the spatial dependence of the original data.

### Mapping

Mapping the (crude) odds of having anti-HAV antibodies (IgG and IgM) was done using binomial generalized additive models (GAM) with the logit link function. GAM is a class of generalized linear models, where the fixed coefficients of the explanatory variables are replaced by smooth functions [13, 14]. In our case, the explanatory variables were a bidimensional smooth function of the geographical coordinates. The formula takes the form:

$$ logit\ \left({y}_i\right) = {\beta}_0 + f\ \left( nort{h}_i,eas{t}_i\right) + {e}_i $$

where *y*_*i*_ is the response variable (positivity for serology), the function *f (north*_*i*_*,east*_*i*_*)* is a smooth function of geographic coordinates and *e*_*i*_ are the residuals. The model was fitted using library mgcv of R software. The resulting maps display the spatial localization of areas with odds of having a positive serology for HAV in Assis Brazil in 2011. Maps showing the spatial localization of social inequities were also constructed. In all maps, the black line separates regions with odds greater and less than two. White contours indicate areas with odds significantly less than the map average (p-value < 0.10), while black contours indicate areas with odds significantly greater than the map average, that is, high-risk areas.

### Ethical considerations

The authors assert that all procedures contributing to this work comply with the ethical standards of the relevant national and institutional committees on human experimentation and with the Helsinki Declaration of 1975, as revised in 2008. The study was approved by the Ethics Committee for Research with Human Beings at the Federal University of Acre. We obtained informed consent from the legal guardian of each participant prior to the study.

## Results

The census performed by the field team identified 364 eligible children in 2011 living in the urban area of the city. All parents and guardians of the eligible participants agreed to let their children participate in the study and in the interview, but not all parents and guardians agreed to have a blood sample collected. Samples were collected from 312 children (85.70 % of the eligible children). Those who did not provide a blood sample were excluded from the analysis. No significant differences *(p*-value > 0.05) were detected for most demographic, socioeconomic, and environmental parameters between subjects who provided a sample and those who did not provide such samples. However, a few differences must be noted. The children that were not tested were similar to those who were, except for their ages, the type of toilet available in their homes, the type of house in which they lived, and the type of street on which they lived. They tended to be younger (75.50 % were younger than 2 years of age, *p* <0.001), living in brick houses (20.80 %, p < 0.045) with flushing toilets (69.80 %, *p* < 0.036), and on paved or unpaved streets (96.20 %, *p* < 0.028) more frequently than those not tested.

Therefore, it is possible that the prevalence of children with detectable antibodies against HAV was slightly overestimated because untested children were younger and living under more favorable conditions.

### Epidemiological characteristics of the study population

Epidemiological characteristics of children that had their blood tested for HAV antibodies are depicted in Table 1.

**Table 1:** Prevalence and crude odds ratio for having a positive serology for Hepatitis A virus in children from 12 to 59 months old. Assis Brasil, 2011

*N* = 312	Number	% hepatitis A	unOR	CI 95 %	*P* value***
*Block 1:Socio-economic*					
*Receipt of benefits*					
No	208	13.94 %	1		
Yes	104	21.15 %	1.66	0.9–3.06	0.107
*Possession of household*					
Owned	203	12.80 %	1		
Not owned	109	22.93 %	2.03	1.1–3.72	0.023
*Years of maternal schooling*					
More than four years	191	12.82 %	1		
Four or less years	121	21.48 %	1.82	0.99–3.33	0.053
*Household stipend*					
More than ½ minimum wage	237	15.18 %	1		
Less or equal to ½ minimum wage	54	20.37 %	1.43	0.67–3.03	0.352
*Block 2: Domestic and peri-domestic environment*					
*Type of household construction*					
Brick walls	34	20.58 %	1		
Wooden walls or another material	278	15.82 %	0.73	0.3–1.77	0.48
*House floor*					
Cement. brick ceramic tile	80	10.00 %	1		
Wood or land	232	18.53 %	2.05	0.92–4.57	0.08
*Is the household located in a street?*					
Yes	266	12.40 %	1		
No	46	39.13 %	4.54	2.26–9.1	<0.001
*Susceptibility to flooding during rain*					
No	170	12.35 %	1		
Yes	141	20.56 %	1.84	1.3–39.0	0.052
*Presence of electric power*					
Yes	299	15.05 %	1		
No	13	46.15 %	4.84	1.55–15.06	0.007
*Number of person per room*	312	16.66 %	1.23	1.11–1.36	<0.001
*Block 3: Sanitary conditions and water quality*					
*Type of toilet*					
Flushed toilet	170	12.35 %	1		
Latrine or no toilet	142	21.12 %	1.9	1.03–3.49	0.039
*Presence of open sewage near house*					
No	169	14.79 %	1		
Yes	143	18.18 %	1.28	0.7–2.33	0.421
*Public waste collection*					
No	25	20.00 %	1		
Yes	287	16.02 %	0.76	0.27–2.14	0.607
*Water supply from public system*					
No	35	0.02 %	1		
Yes	277	22.02 %	7.49	1.00–56.00	0.05
*Water supply from wells*					
No	270	17.77 %	1		
Yes	42	7.14 %	0.36	0.11–1.2	0.095
*Piped water supply inside the household*					
Yes	195	12.82 %	1		
No	117	22.22 %	1.94	1.06–3.56	0.031
*Mineral water for drinking*					
No	184	15.76 %			
Yes	128	17.18 %	1.11	0.60–2.03	0.738
*Treatment of drinking water with chlorine*					
No	278	15.82 %	1		
Yes	34	20.58 %	1.38	0.57–3.36	0.48
*Block 4 – Characteristics of the child*					
*Gender*					
Male	157	13.37 %	1		
Female	155	19.35 %	1.55	0.85–2.86	0.155
*Age (in months)*					
12 to 23	71	11.26 %	1		
24 to 35	82	14.63 %	1.35	0.52–3.52	0.539
35 to 48	72	13.88 %	1.27	0.47–3.43	0.637
48 to 59	87	24.13 %	2.51	1.03–6.07	0.042
*Ethnicity*					
Non-indigenous	263	12.54 %	1		
Indigenous	45	40.00 %	4.73	2.35–9.51	<0.001

*** Wald test

### House characteristics and sanitation conditions

Most of the children lived in wooden houses that their parents owned, with wooden floors, electric power, and public waste collection. Approximately 14.70 % of the children lived in houses that were not accessible from the street, but only by walking among other houses. An expressive number of children was living in poor sanitation conditions: 45.50 % of them lived in houses with latrines or no toilets, 45.80 % were living in environments with direct contact with open sewage and 45.30 % lived in houses subjected to flooding on rainy days (Table 1). The average number of people per room was 3.53 (minimum 0.75, maximum 13.00).

### Water source and usage

The percentage of children with a public water supply at home was 88.70 %. Only 62.20 % of children had a piped water supply inside the household. The water supply at home also came from wells (13.40 %), mineral water (41.00 %), and others sources (3.80 %), such as pitfalls, river, rain, and cisterns.

### Socioeconomic and demographic features

The proportion of male and female children was the same, and 14.40 % of the children were from indigenous ethnicity, while 18.60 % were Caucasians and 67.00 % were black or “pardos’ (the offspring between Caucasian and black people). One third of the children benefited from the governmental Bolsa Familia Program, which distributes a monthly income for those classified as poor under government criteria. Still, 18.50 % belonged to families for which the monthly stipend was less than half of minimum wage (approximately $120). Of the mothers and female caregivers, 38.80 % attended school for fewer than four years, and a portion were illiterate (13.70 %) (Table 1).

### Prevalence of anti-HAV total antibodies and associated factors

**Fig. 1:**
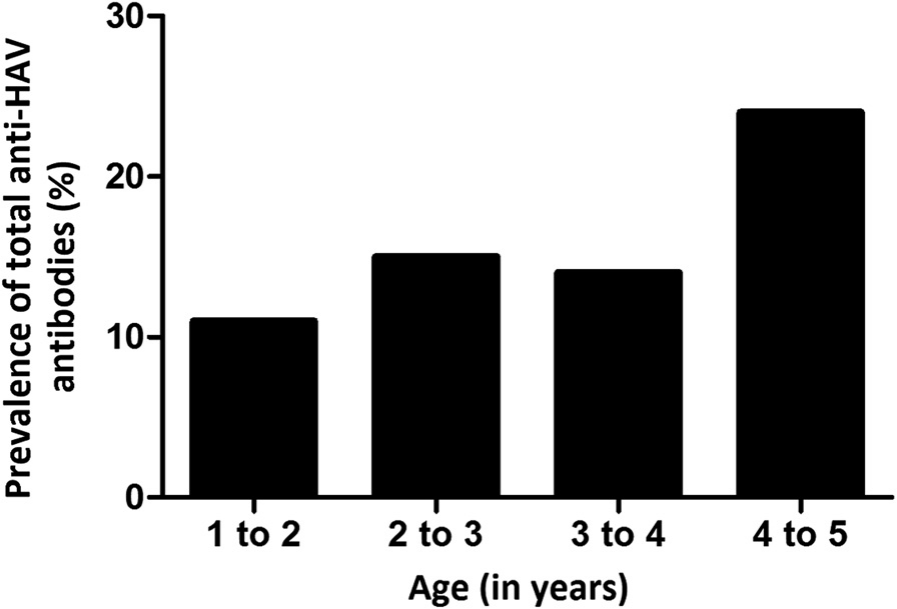
Prevalence of total anti-HAV antibodies (%) by age (in years), Assis Brasil, 2011

The overall prevalence of seropositive children was 16.66 %. Table 1 describes the prevalence according to socioeconomic, demographic, and sanitary conditions, highlighting those with crude odds ratio < 20.00 %. Figure 1 shows the variation of prevalence by age. Combining these variables into a multiple logistic model (the LOG model), only four variables remained significant (Table 2): child ethnicity, access to water supply from a public system, whether or not the house was located on a street, and child age (after dichotomizing as greater or less than 4 years-old). Overall, according to this model, children between 4 and 5 years of age were more likely to have positive serologies than younger children [adjusted Odds Ratio (aOR) 2.39, 95 % Confidence Interval (CI) 1.21–4.72]. Indigenous children were also more likely to have detectable antibodies against HAV than non-indigenous children (aOR 3.24, 95 % CI 1.44–7.31). Using water from the public system at home was associated with the presence of antibodies against HAV, with an adjusted odds ratio of 8.12 (95 % CI 1.06–62.12). Finally, living in a house that was not directly located on a street (e.g., located at the back of another house or in areas of illegal or unplanned occupation) was associated with higher odds of positive serology (aOR = 2.39, 95 % CI 1.21–4.72). No evidence of spatial autocorrelation was found the residuals of the final model, suggesting that the covariates were sufficient to account for the spatial structure in the odds of having anti-HAV antibodies (Additional file 1: Figure S1).

**Table 2:** Adjusted odds ratio for having a positive serology for Hepatitis A virus in children from 1 to 5 years old. Assis Brasil. 2011

Variables (*N* = 312)	unOR	CI 95 %	aOR	CI 95 %	*P* value
*Child ethnicity*					
Non-indigenous	1				
Indigenous	4.73	2.35–9.51	3.27	1.45–7.38	0.004
*Is the household located in a street?*					
Yes	1				
No	4.54	2.26–9.1	3.48	1.54–7.87	0.003
*Water supply from public system*					
No	1				
Yes	7.49	1.00–56.00	8.17	1.07–62.53	0.043
Age					
< 4 years	1				
4 to 5 years	2.07	1.11–3.86	2.43	1.23–4.79	0.011

Because being of indigenous origin was highly associated with having a positive serology for the hepatitis A virus, analysis was performed regarding which social inequity was associated with indigenous ethnicity using the same methodology described previously. Indigenous children had higher odds of living in unowned, crowded houses with wooden floors, without electricity, and located on unplanned or absent streets. They tended to have mothers with less education and belong to families with lower monthly income than non-indigenous children. Ethnicity was also associated with unfavorable sanitary conditions because indigenous children were living in houses with less access to flushing toilets and with more exposure to open sewage. They also tended to have less access to piped water and mineral water within the household and add chlorine to their water more frequently (Additional file 2: Table S1).

### Spatial distribution of positive serology for HAV

**Fig. 2:**
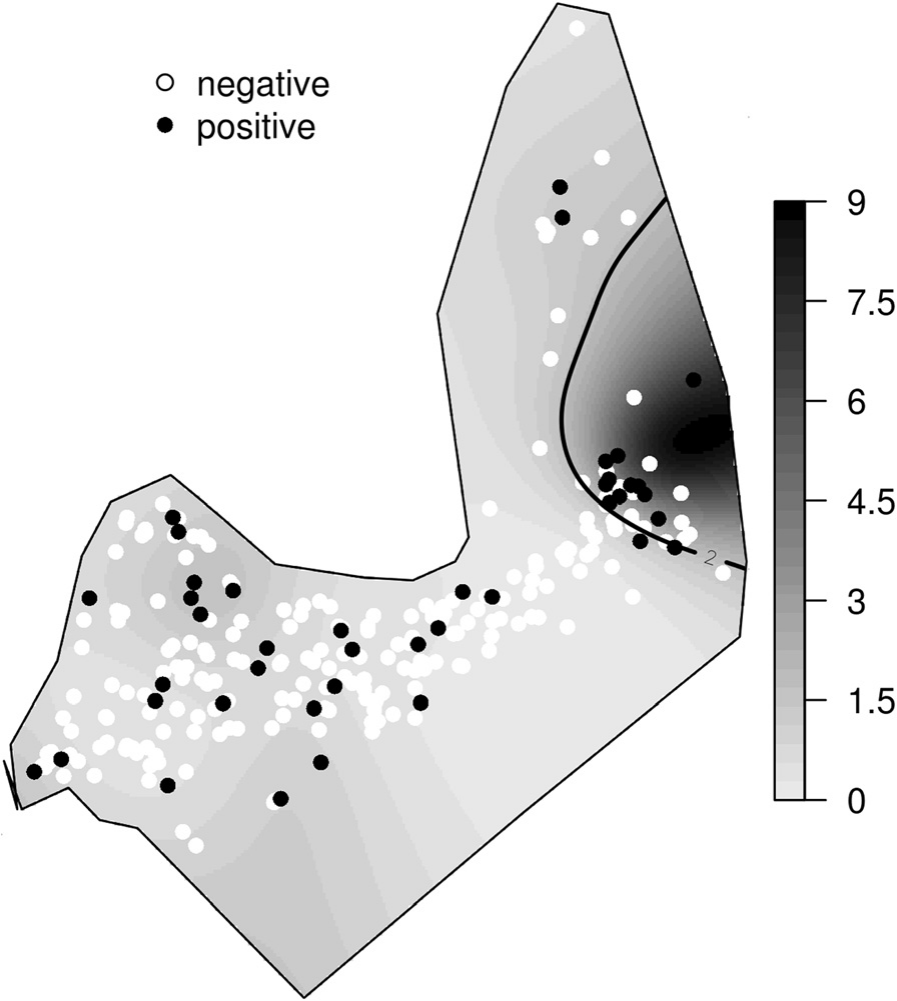
Spatial odds ratio for the prevalence of total anti-HAV antibodies in Assis Brasil for the year of 2011. Crude odds ratio surface estimated by a GAM model. Positive cases are represented in black dots and negative in white dots. Odds ratio values are represented in a gray scale, from 0 to 8.33. Black lines limitate the area where odds ratio is higher than 2. A hotspot of anti-HAV-positive children is present in the new easternmost area

Figure 2 shows the estimated odds ratio surface according to generalized additive model for the prevalence of total anti-HAV antibodies in Assis Brasil for 2011. Darker areas indicate a greater odds ratio. A hotspot of anti-HAV-positive children was found in the new easternmost area of the town. (Fig. 2).

### Spatial distribution of social inequities

Spatial analysis detected a statistically significant spatial cluster for the following socioeconomic and environmental variables: type of house floor; susceptibility of house flooding during rain; type of toilet; access to mineral water and water from the public system; possession of household; maternal education; and ethnicity.

**Fig. 3:**
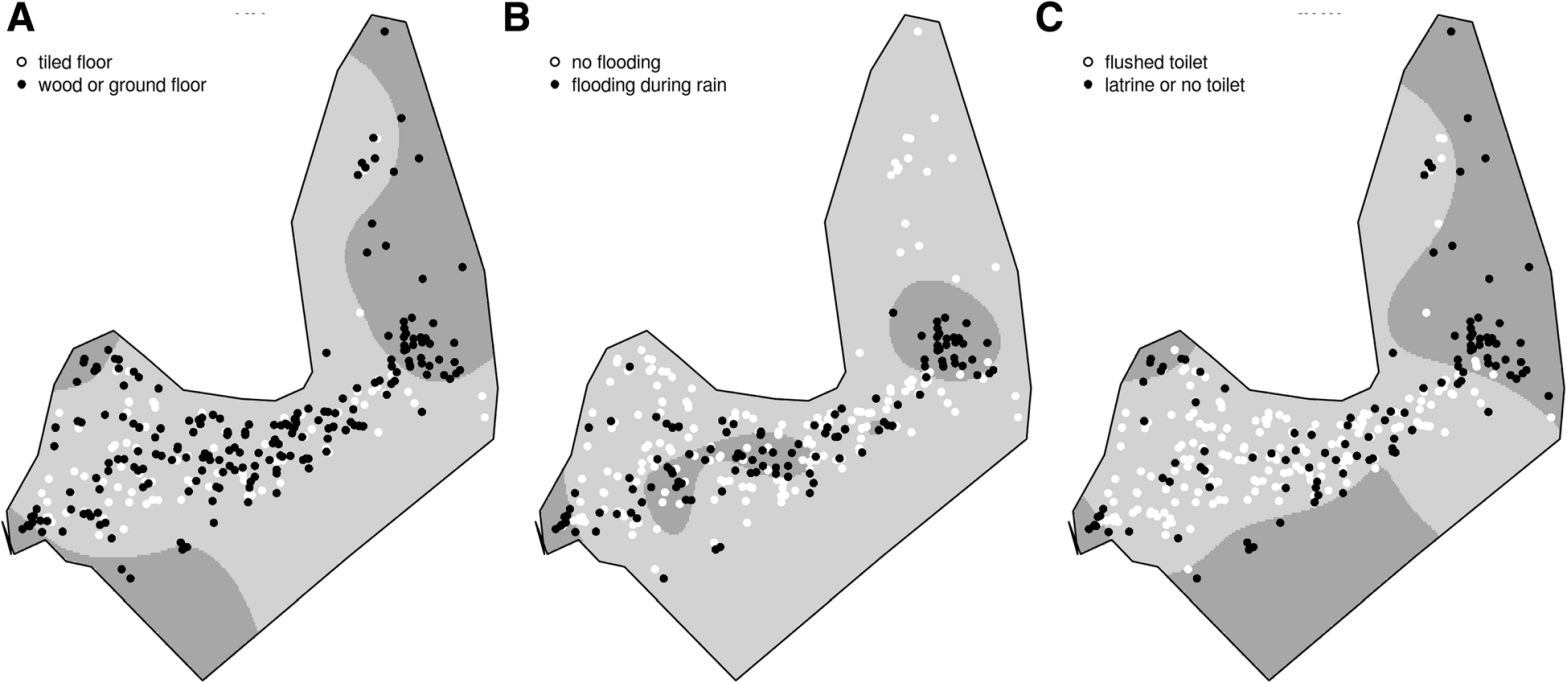
Spatial odds ratio for the prevalence of (**a**) owned household, (**b**) maternal schooling, and (**c**) child ethnicity. Crude odds ratio surface estimated by a GAM model. Black dots represents (**a**) not owned households, (**b**) maternal schooling less than four years, (**c**) indigenous ethnicities, and white dots represents (**a**) owned households, (**b**) maternal schooling 4 or more years, (**c**) non indigenous ethnicity. The darker areas are where odds ratio is higher than 2

Unfavorable housing conditions, such as having a wood floor (Fig. 3a), household flooding during rain (Fig. 3b), and absence of flushing toilets at home (Fig. 3c), were clustering in the easternmost area of the town, similar to the anti-HAV antibodies clustering area. These unfavorable housing conditions also clustered in the westernmost portion of the city (Fig. 3).

**Fig. 4:**
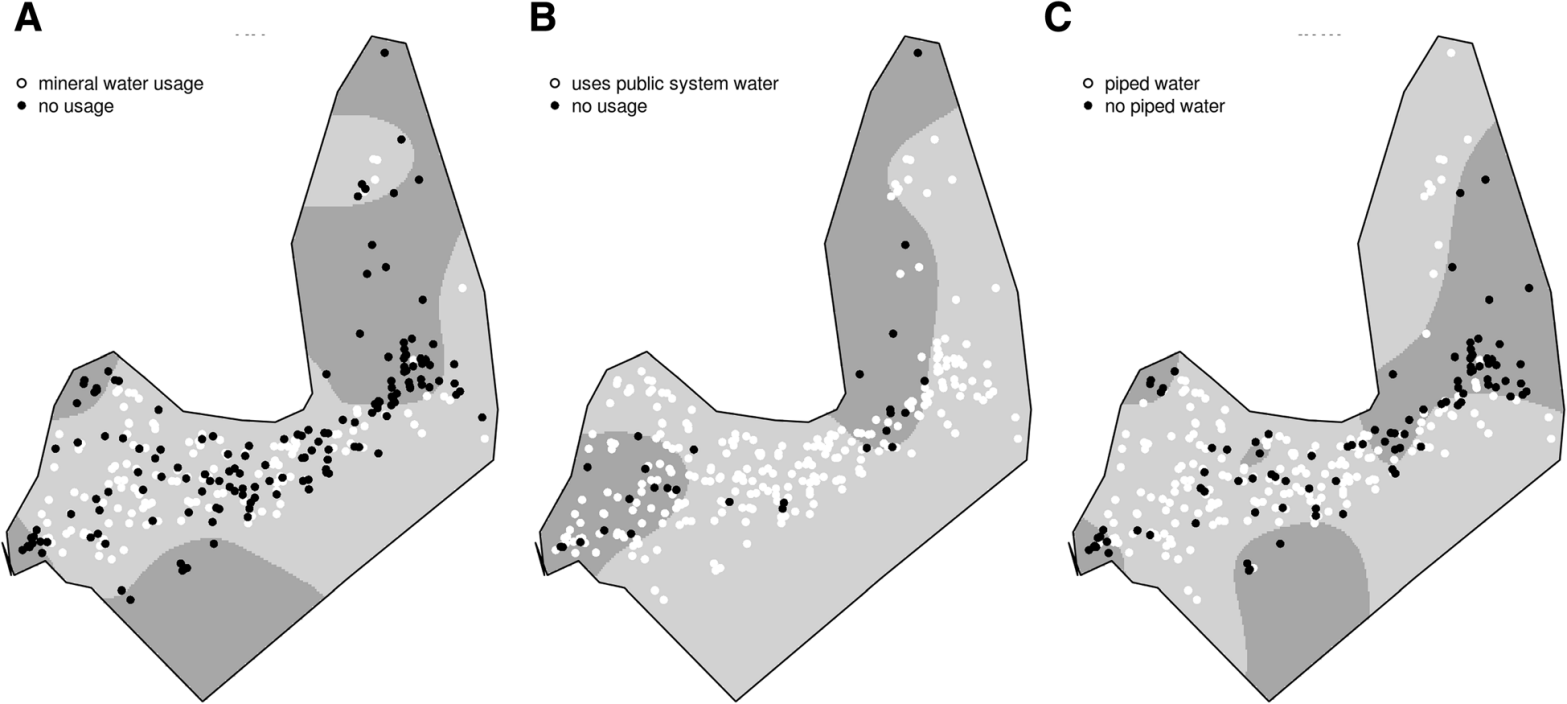
Spatial odds ratio for the prevalence of (**a**) type of toilet, (**b**) house floor (**c**) household susceptibility to flooding during rain. Crude odds ratio surface estimated by a GAM model. Black dots represents (**a**) latrine or no toilet, (**b**) wood or ground floor, (**c**) flooding during rain, and white dots represents (**a**) flushed toilet, (**b**) tiled floor, (**c**) no flooding during rain. The darker areas are where odds ratio is higher than 2

Lack of access to mineral water and to piped water inside the household had greater spatial odds in the same regions: the easternmost part of the city (similar to the hotspot of anti-HAV positive children) and the westernmost area of the city (Fig. 4a and c). Households that did not use water from the public system were concentrated in the Northern region of the city and a large portion of the western part of the city (Fig. 4b). When comparing results in Figs. 1 and 3b, we can note that most of the houses included in the hotspot for seropositivity for HAV antibodies used water from the public system (Fig. 4).

**Fig. 5:**
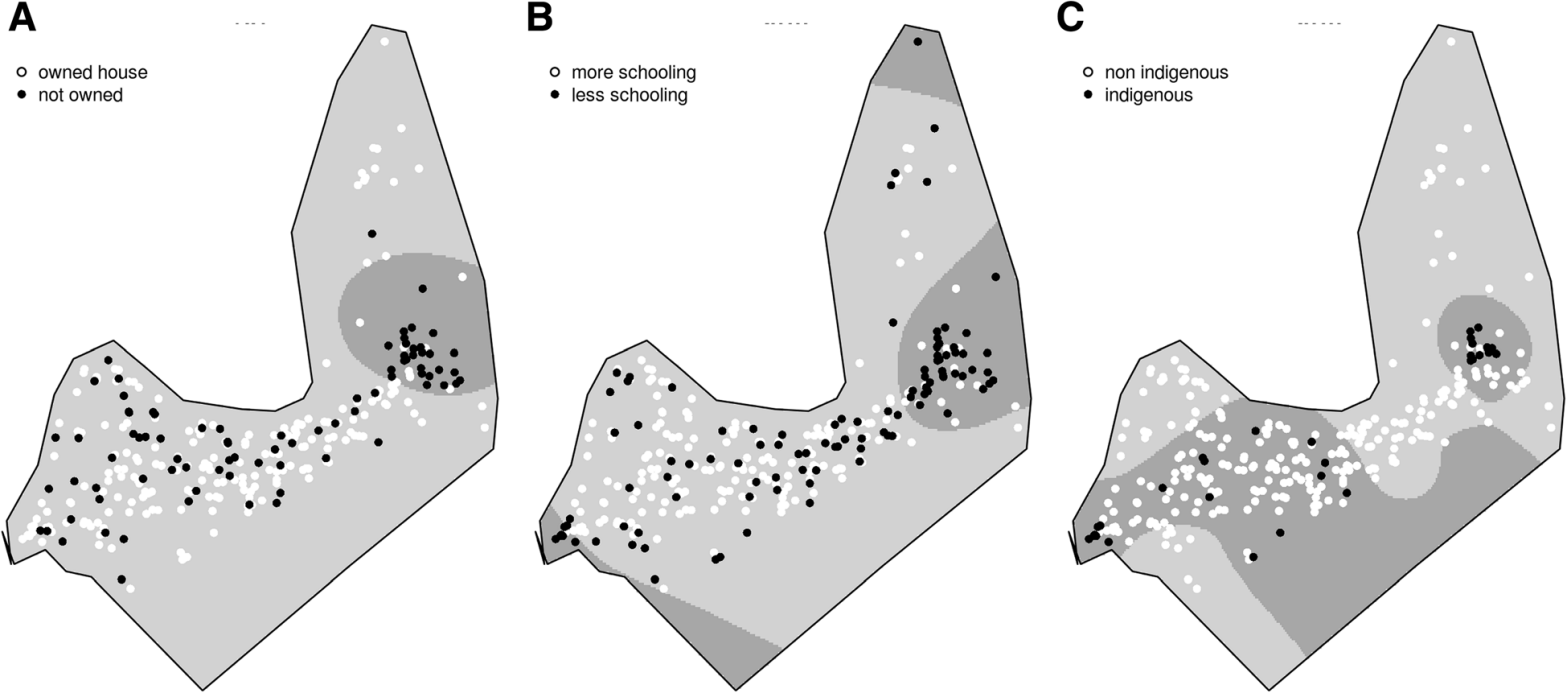
Spatial odds ratio for the prevalence of (**a**) access to mineral water for drinking, (**b**) usage of water from the public system (**c**) piped water inside the household. Crude odds ratio surface estimated by a GAM model. Black dots represents (**a**) no access to mineral water for drinking, (**b**) no use of to water from the public system, (**c**) no piped water inside the household, and white dots represents (**a**) use of mineral water for drinking, (**b**) access to water from public system, (**c**) no piped water inside the household. The darker areas are where odds ratio is higher than 2

Poor socioeconomic conditions measured by not owning a house and low maternal education had significant higher spatial odds in the westernmost area of the city (Fig. 5a and b), similar to the spatial hotspot of anti-HAV positive children (Fig. 5).

Children from indigenous origin (born from an indigenous mother or father) were more likely to live in the easternmost or the westernmost areas of the city (Fig. 5c). This spatial distribution of indigenous children is very similar to the hotspots for HAV-positive children—low maternal scholarity, not having access to piped water and mineral water at home, lack of flushing toilets, and other unfavorable environmental conditions, such as household flooding and wood floors.

No statistically significant hotspots were detected for all other variables tested, such as sex, child age, house situated on a street, household monthly income, presence of electricity and public waste collection, and presence of open sewage near the house.

## Discussion

Seroprevalence for hepatitis A antibodies varies according to age strata and region. In Assis Brasil, seroprevalence for hepatitis A antibodies in children 1 to 4 years of age was 16.67 %. Bensabath et al. [15] studied two neighboring cities in the Amazon, one in the state of Acre and the other in the state of Amazonas, and found very different seroprevalences between 1979 and 1984. Boca do Acre (Amazonas) had higher seroprevalences (45.80 % and 59.40 % in age strata 0 to 2 years of age and 3 to 4 years of age, respectively), while Sena Madureira had much lower seroprevalences (13.80 % and 37.50 % for the same age strata) [15]. In Labrea, another city neighboring Boca do Acre, seroprevalence for anti-HAV IgG ranged from 32.30 % in children 1 year of age to 34.30 % to children 4 years of age [3] in 2005. Therefore, the seroprevalence for anti-HAV antibodies found in our study is similar to other neighboring cities.

However, higher seroprevalence rates have been found in other Amazonian populations. In 1998, after a hepatitis outbreak, Assis et al. [5] found a seroprevalence for IgG-anti-HAV between 62.50 % and 76.90 % in children 3 to 4 years of age in the Amazon. Laffer et al. [16] found a seroprevalence of 93.70 % in indigenous children 1 to 5 years of age from Xingu National Park in 2001, which is much higher than the prevalence reported in Acre.

Average seroprevalence in the Northern region of Brazil (where the Amazon is located) is higher than in the rest of the country. Vitral et al. [17] found seroprevalences of 25.90 % for the Northern region as compared to a seroprevalence of 10.00 % for the Southwest region, while Clemens et al. [1] reported seroprevalences of 92.80 % and 55.70 %, respectively. In fact, seroprevalence in children 1 to 4 years of age living in Rio de Janeiro (Southeast Brazil) was reported to be 5.01 % to 27.46 % [18]. In a study in South Brazil (Curitiba), total anti-HAV seroprevalence was 3 % in children 1 to 4 years of age [19], and the seroprevalence of anti-HAV IgG in Santos was 2.50 % in daycare facilities and 5.50 % in kindergarten children [20]. These are much lower seroprevalences than those reported in Amazonian studies and lower than rates found in Rio de Janeiro; however, they were not population-based studies.

World Health Organization (WHO) reports for children 1 to 4 years of age in other areas of the world show seroprevalences lower than 5.00 % in North America, Western Europe, Australasia and high-income areas of the Asia Pacific [21]. Seroprevalence rates are between 50.00 % and 75.00 % in the Andean region; Central Latin America; West, South, and East Subsaharian Africa; and South Asia, and rates are between 15.00 % and 50.00 % in Eastern Europe and the remaining areas of Asia, Latin America, Africa, and the Middle East [21], suggesting that hepatitis A transmission is also linked to socio-economic development in the rest of the world.

One important issue in the transmission of hepatitis A is age at which children are infected and whether or not children should be vaccinated. This seems to vary according to transmission levels. In a national survey performed in Brazil in 2010, seroprevalence of 30 % was found in children 10 to 14 years of age, while in areas of intermediate prevalence, the same rate was found in children between 5 and 9 years of age [22]. Vitral et al. [17] has shown declining seroprevalence for hepatitis A virus in Brazilian capitals, probably as the result of improved sanitary conditions and water treatment. The authors discuss that these decreased levels of transmission at some areas without proper immunization could result in shifting of infection to older age strata, resulting in more symptomatic and fatal cases [17]. Modeling strategies have shown that, in areas of intermediate endemicity, the risk for acquiring hepatitis A increases until 25 years of age, while at low-transmission settings, the force of infection remains the same in all age groups [22]. In view of this recent shift in hepatitis A epidemiology, the Brazilian government has just introduced the hepatitis A vaccine into the Brazilian National Immunization Program, targeting it toward children between 12 and 23 months of age [23]. This strategy is expected to result in a 64.00 % reduction in the number of icteric cases and a 59.00 % reduction in deaths caused by hepatitis A, at a cost of 7.23 U.S. dollars per dose [24].

Positive serology for hepatitis A was associated with being of indigenous origin, having a house not located on a street, using water from a public system, and being older than 4 years of age. Brazil is a country of ethnic diversity and it is widely known that social inequities are frequently associated with ethnicity. At the same time, ethnic groups have a heterogeneous spatial distribution because of past migratory movements. Indigenous ethnicity is widespread in Brazil but concentrates in the Northern region. Caucasians predominate in the Southern and Southeastern part of the country, Blacks and people of Asian origin predominate in Southeastern Brazil, and “Pardos” (the offspring of a Caucasian and black couple) predominate in the Northeastern part of Brazil [25]. Therefore, it is common to have population-based studies with multiethnic composition. In Assis Brasil, this minority group comprised 35.3 % of the positive cases and, at the same time, this ethnic group was associated with most of the socioeconomic inequities that were evaluated (low maternal education, latrine or no toilet, lack of piped water, presence of open sewage near the house, and lack of access to mineral water).

Several studies have already shown the social vulnerability of the indigenous population in Brazil [26]. Coimbra et al. [27] report poor sanitation and living conditions for indigenous populations, which can explain why seroprevalence for hepatitis A is high. These poor sanitation conditions, which are worse in the Amazon, include very low availability of latrines inside the household (0.60 %), lack of sewage treatment in 91.00 % of the indigenous households (while 90.00 % of the non-indigenous households in Brazil do have sewage treatment), and lack of waste collection in 79.00 % of the indigenous areas. Indigenous people also face inequities regarding water treatment; less than 30.00 % of indigenous households have access to water from the public system and 40.00 % of them collect water from wells, lakes, or rivers. Pena and Heller [28] also found a great proportion of Brazilian indigenous children (between 75.00 % and 100.00 %) without access to water from the public system or without access to sanitary installations.

Recent nationwide research about health in the indigenous population identified that children in this ethnic group have much higher prevalences of anemia (66.20 %) [29] and undernutrition (40.80 %) [30] than non-indigenous children (20.90 % and 14.70 %, respectively) living in the Amazon [27]. Nunes et al. [31] showed seroprevalence of 96.20 % in indigenous children 1 to 4 years of age and 98.00 % in the general indigenous population of Altamira after an outbreak in the eastern Brazilian Amazon, which is much higher than seroprevalence for non-indigenous Amazonian children. Laffer et al. [16] also reported similar seroprevalence in indigenous tribes of Xingu (97.70 %). Bialek et al. (2004) [32] also showed a high incidence of hepatitis A cases in indigenous people in the US and Alaska, in areas where the vaccine was not fully available, confirming the vulnerability of native populations. Therefore, the indigenous ethnic group is exposed to several socioeconomic inequities, both in and outside of the Amazon, and, at the same time, present higher prevalence of several infectious diseases, most of them related to these unfavorable socioeconomic conditions [26].

In the present study, water usage from the public system was associated with positive serology for anti-HAV antibodies. It is important to note that, in the Amazon, not all water distributed by the governmental public system receives proper treatment, and our results suggest that the quality of water distributed by the municipality was not adequate. There is no specific data on water treatment for Assis Brasil, but recent data for sanitation conditions in the capital city of Rio Branco showed that only 27.00 % of all the sewage is collected and only 3.00 % of the water is treated [33]. It is possible that because of poor sanitation and lack of sewage treatment in the city, contamination of local water sources by fecal coliforms may be frequent and, eventually, the hepatitis A virus may be present in the untreated water consumed by the population. An association between other water sources (such as rivers, lakes, and streams) and a positive serology (probably due to sample size limitations) was not detected.

The quality of drinking water and water used for domestic purposes has been implicated with the transmission of hepatitis A in several other studies. Zago-Gomes et al. [34] have found decreased seroprevalence in children 4 to 14 years of age who had access to filtered water in Midwestern Brazil. Vitral et al. [17] also found association between the use of filtered water and decreased seroprevalence for hepatitis A in children less than 18 years of age in different cities in Brazil. The same association was found by Almeida et al. [18] in the city of Rio de Janeiro. A study performed between 2004 and 2005 in the Amazon detected hepatitis A viral load in 92.00 % of water samples from streams collected in the city of Manaus, which is one of the most urbanized cities in the Brazilian Amazon [35]. This result suggests the contamination of rivers and streams with untreated sewage. Several outbreaks of acute hepatitis A have been shown to be associated with contact with contaminated water [36, 37]. All of these studies demonstrated that the quality of water and how it is regularly used is a major question in hepatitis A transmission, regardless of how exposure to water of poor quality was measured.

Since the last decade, bottled water that is sold as “mineral water” or “water extracted from deep soil fountains” has been extensively commercialized in Brazil and has started to be sold in the Amazon as well. It has a higher cost than water from the public system or from wells and it is supposedly of high quality; therefore, it could act as a protective factor from contact with the hepatitis A virus. Although no statistically significant association between access to bottled water (or “mineral” water, as it is called in Brazil) and hepatitis A cases was found in this study, consumption of this type of water was spatially concentrated in areas with low seroprevalence of anti-HAV antibodies.

Some variables were associated with the presence of anti-HAV antibodies in the unadjusted analysis. Among them are variables that are proxies of socioeconomic conditions, such as maternal education, type of house floor, lack of electricity, and not owning a house. After adjustment for other factors, they were no longer statistically significant, but three of them (low maternal education, wood or ground floor, and not owning a house) had a significant spatial cluster that was superimposed on the spatial cluster of HAV cases, suggesting that these two socioeconomic inequities occur more frequently in Assis Brasil in households where HAV cases are concentrated. Ciaccia et al. [20] found an association between presence of anti-HAV antibodies in children up to 18 years of age and low maternal education in Southern Brazil, but it is possible that low maternal education was a proxy of low socioeconomic conditions in this study, as it was in Assis Brasil.

Three other variables were associated with the presence of anti-HAV antibodies in the univariate analysis and had a spatial clustering of cases. These include type of toilet, presence of piped water inside the household, and susceptibility of the household to flooding during rain. These indicate unfavorable environmental conditions that may be associated with facilitated transmission of the hepatitis A virus. Gomes et al. [38] and Ximenes et al. [39] also showed that lack of piped water supply at home was related to increased seroprevalence of anti-HAV antibodies in children and adolescents in Northeastern and Midwestern Brazil, respectively. Contact with open sewage around the house and lack of toilets or latrines have also been implicated as associated factors in other studies [18, 40]. Living in households that are not located on a street may represent an unfavorable environmental condition because public services such as sanitation, piped water, and waste collection may not be available.

This study has two limitations. Seroprevalence was calculated using total anti-HAV antibodies, and the infection may have occurred in the past while variables are being measured in the present. However, because the age group is 1 to 4 years of age, it is probable that this had little effect in most of the variables measured, which are not expected to change much over a short period of time. The children that were not tested were younger and living in more favorable conditions than those tested and, therefore, it is possible that the prevalence of children with detectable antibodies against HAV was slightly overestimated and the size of the association between the mentioned variables and seroprevalence was overestimated as well. However, these limitations do not affect the overall quality of the study.

## Conclusion

The results of this study show that, even today, risk factors for hepatitis A transmission continue to be related to sanitation issues. Sewage treatment and implementation of septic tanks can prevent contamination of rivers, wells, and water from the public system. In fact, the study of de Paula et al. [35] demonstrating the presence of hepatitis A virus RNA in urban streams shows that lack of sewage treatment is still a major issue in the Amazon. Proper water treatment can also reduce transmission. Boiling water before use is an inexpensive treatment and can be very effective against hepatitis A [41]. The use of chlorine in the drinking water is adopted worldwide to protect against the infection of HAV. The disinfection with chlorine has low-to-moderate effectiveness against the protozoa *Giardia lamblia* and high effectiveness against bacteria and viruses (including HAV) [41]. To be effective, though, the optimal dilution must be used, and sometimes during domestic use, these optimal conditions are not achieved. Therefore, public water treatment facilities should be made available in areas where hepatitis A virus can be present.

Other major investments that are needed are continuous sanitation programs with sewage treatment and building of latrines or flushing toilets that will prevent contact with contaminated sewage. Education in health is also important for reducing the transmission of the hepatitis A virus and other infectious diseases. In conclusion, hepatitis A transmission is still associated with socioeconomic and environmental inequities.

## Additional files

Additional file 1: Figure S1. Spline correlograms, with 95 % pointwise bootstrap confidence intervals, of the Pearson residuals from the logistic regression models applied to “having anti-HAV antibodies”: (A) model with no covariates (null model) showing spatial correlation at distances less than ca 100 m; and (B) model with all covariates of the final model, indicating that no spatial structure remained in the residuals after the introduction of the explanatory variables. (TIFF 21 kb) Additional file 2: Table S1. Social inequities associated with being of indigenous ethnicity in children 12to 59 months old. Assis Brasil, 2011. (DOCX 19 kb)
