# Supplementary figures and images for: Socioeconomic inequities and hepatitis A virus infection in Western Brazilian Amazonian children: spatial distribution and associated factors
Source: BMC Infect Dis. 2015 Oct 16;15:428. doi: 10.1186/s12879-015-1164-9 (PMC4608050; doi:10.1186/s12879-015-1164-9)

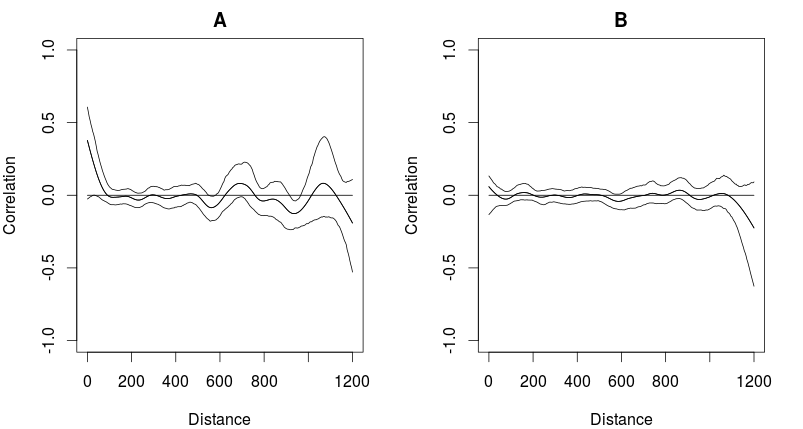

Supplement: Additional file 1: Figure S1. — Spline correlograms, with 95 % pointwise bootstrap confidence intervals, of the Pearson residuals from the logistic regression models applied to “having anti-HAV antibodies”: (A) model with no covariates (null model) showing spatial correlation at distances less than ca 100 m; and (B) model with all covariates of the final model, indicating that no spatial structure remained in the residuals after the introduction of the explanatory variables. (TIFF 21 kb) [file 12879_2015_1164_MOESM1_ESM.tiff]
