# Supplementary material for: Socioeconomic inequities and hepatitis A virus infection in Western Brazilian Amazonian children: spatial distribution and associated factors
Source: BMC Infect Dis. 2015 Oct 16;15:428. doi: 10.1186/s12879-015-1164-9 (PMC4608050; doi:10.1186/s12879-015-1164-9)
Supplement: Additional file 2: Table S1. — Social inequities associated with being of indigenous ethnicity in children 12to 59 months old. Assis Brasil, 2011. (DOCX 19 kb) [file 12879_2015_1164_MOESM2_ESM.docx]

**Additional file 2: Table S1**–Social inequities associated with being of indigenous ethnicity in children 12to 59 months old. Assis Brasil, 2011.

| **N = 312** | **N** | **% indigenous children** | **unOR** | **CI 95%** | **P value***** |
| --- | --- | --- | --- | --- | --- |
| ***Block 1:Socio-economic*** |  |  |  |  |  |
| ***Receipt of benefits*** |  |  |  |  |  |
| No | 208 | 6.73 | 1 |  |  |
| Yes | 104 | 29.80 | 5.88 | 2.96-11.69 | **< 0.001** |
| ***Possession of household*** |  |  |  |  |  |
| Owned | 203 | 8.86 | 1 |  |  |
| Not owned | 109 | 24.77 | 3.37 | 1.76-6.46 | **< 0.001** |
| ***Years of maternal schooling*** |  |  |  |  |  |
| Four or less years | 121 | 29.75 | 1 |  |  |
| More than four years | 191 | 4.71 | 0.12 | 0.05-0.25 | **< 0.001** |
| ***Household stipend*** |  |  |  |  |  |
| Less or equal to ½ minimum wage | 54 | 29.62 | 1 |  |  |
| More than ½ minimum wage | 237 | 10.97 | 0.29 | 0.14-0.60 | **< 0.001** |
| ***Block 2: Domestic and peri-domestic environment*** |  |  |  |  |  |
| ***House floor*** |  |  |  |  |  |
| Tile floor | 80 | 3.75 | 0.3 | 0.015-0.057 | **<0.001** |
| Wood or ground floor | 232 | 18.10 | 1 |  |  |
| ***Is the household located in a street?*** |  |  |  |  |  |
| Yes | 266 | 9.39 | 1 |  |  |
| No | 46 | 43.47 | 7.42 | 3.63-15.14 | **< 0.001** |
| ***Susceptibility to ﬂooding during rain*** |  |  |  |  |  |
| Yes | 141 | 27.65 | 10.45 | 4.27-25.56 | **< 0.001** |
| No | 170 | 3.52 | 1 |  |  |
| ***Presence of electric power*** |  |  |  |  |  |
| No | 13 | 69.23 | 1 |  |  |
| Yes | 299 | 12.04 | 0.06 | 0.02-0.21 | **< 0.001** |
| ***Persons per room*** |  |  |  |  |  |
| *<4* | *198* | 4.08 |  |  |  |
| *>=4* | *114* | 33.03 | 11.59 | 5.16-26.05 | **< 0.001** |
| ***Block 3: Sanitary conditions and water quality*** |  |  |  |  |  |
| ***Type of toilet*** |  |  |  |  |  |
| Flushed toilet | 170 | 2.94 | 1 |  |  |
| Latrine or no toilet | 142 | 28.16 | 12.94 | 4.95-33.86 | **< 0.001** |
| ***Presence of open sewage near house*** |  |  |  |  |  |
| No | 169 | 6.50 | 1 |  |  |
| Yes | 143 | 23.77 | 4.48 | 2.18-9.23 | **< 0.001** |
| ***Domestic water supply from public system*** |  |  |  |  |  |
| No | 35 | 14.28 | 1 |  |  |
| Yes | 277 | 14.44 | 1.01 | 0.37-2.76 | 0.98 |
| ***Piped water supply inside the household*** |  |  |  |  |  |
| No | 117 | 29.05 | 1 |  |  |
| Yes | 195 | 5.64 | 0.15 | 0.07-0.3 | **<0.001** |
| ***Mineral water for drinking*** |  |  |  |  |  |
| No | 184 | 20.10% | 1 |  |  |
| Yes | 128 | 6.25% | 0.26 | 0.12-0.59 | **0.001** |
| ***Treatment of drinking water with chlorine*** |  |  |  |  |  |
| No | 278 | 12.22 | 1 |  |  |
| Yes | 34 | 32.35 | 3.43 | 1.54-7.66 | **0.003** |

*** Wald test
